# Evolving MCDM Applications Using Hybrid Expert-Based ISM and DEMATEL Models: An Example of Sustainable Ecotourism

**DOI:** 10.1155/2013/751728

**Published:** 2013-12-23

**Authors:** Huan-Ming Chuang, Chien-Ku Lin, Da-Ren Chen, You-Shyang Chen

**Affiliations:** ^1^Department of Information Management, National Yunlin University of Science and Technology, No. 123, University Road, Section 3, Douliou, Yunlin 640, Taiwan; ^2^Department of Information Management, National Taichung University of Science and Technology, No. 129, Section 3, Sanmin Road, North District, Taichung 404, Taiwan; ^3^Department of Information Management, Hwa Hsia Institute of Technology, No. 111, Gong Jhuan Road, Chung Ho District, New Taipei 235, Taiwan

## Abstract

Ecological degradation is an escalating global threat. Increasingly, people are expressing awareness and priority for concerns about environmental problems surrounding them. Environmental protection issues are highlighted. An appropriate information technology tool, the growing popular social network system (virtual community, VC), facilitates public education and engagement with applications for existent problems effectively. Particularly, the exploration of related involvement behavior of VC member engagement is an interesting topic. Nevertheless, member engagement processes comprise interrelated sub-processes that reflect an interactive experience within VCs as well as the value co-creation model. To address the top-focused ecotourism VCs, this study presents an application of a hybrid expert-based ISM model and DEMATEL model based on multi-criteria decision making tools to investigate the complex multidimensional and dynamic nature of member engagement. Our research findings provide insightful managerial implications and suggest that the viral marketing of ecotourism protection is concerned with practitioners and academicians alike.

## 1. Introduction

This section includes the engagement behavior for a virtual community, the importance of environmental concerns, and our research objectives and problems.

As information technology (IT) advances, the words “community” and “network” are commonly referred to as “virtual community, VC.” VC sites formed by the internet offer users the ability to interact with others and exchange information and knowledge of the environment. Various social networking sites are emerging to attract a large number of users to participate in discussions on current issues and trends. Previous studies that indicate the factors that will affect user interest to use social networking sites are mainly focused on their initial beliefs (perceived usefulness and ease of use) as well as the attitudes to explore ideas for their use. Chen [1] claimed that the concept of community network originated from Barnes [2]: an anthropologist discovered the concept from an investigation of the social structure of a Norwegian fishing village. One cannot clearly explain the actual operations of a fishing village by looking at the formal role of social structure, such as identity or status. Instead, one can explain the interactions in a fishing village from looking at the informal role of social structure, such as friends and relatives and how they organize and impact an internal social network. Mitchell [3] argued that social networks show the interrelationship among individuals of a particular society. Pattison [4] believed that social networks are a social organization that collectively gather from the interrelationship among individuals and organizations. Chan et al. [5] claimed that individual behavior and attitudes demonstrate that a particular environment was influenced by the interconnections with other individuals in that social environment. The network connects nodes and nodes together form the structure. The nodes can be individuals, teams, or organizations [6], and its overall structure describes social behavior.

In recent years, development of VC websites has grown rapidly. The use of social networking and their fast growth have become an important trend. According to the Business Next [7] survey of the top 100 Taiwan websites, 25 community sites were selected among to represent 25% of the overall list. After the financial crises of 2008, the VC website development seems to be the most promising and popular business model or trend. This trend is buttressed by social networking sites and microblogs, such as Facebook, Twitter, and Plurk. Users from US President Barack Obama, former GE chief executive Jack Welch, to Dell Computer and Starbucks Coffee, are utilizing this trend [8]. According to a survey by ComScore [9], the number of VC websites has grown at an amazing rate.

How will social networking sites grow to replace other types of sites, such as shopping sites and search engine websites, as users actively become more involved with these activities? Here are some important reasons. (1) Social networking sites bring interpersonal relationships into virtual situations; (2) they create more opportunities for human interaction and real-time sharing of personal information such as pictures, videos, and interactive games; and (3) users can create their own program to share many features that will meet their social, leisure, and entertainment needs. Thus, social networking sites successfully win user interest, which makes it a popular site type. They attract many users in VCs to share information and emotional communication. This free, open yet with hidden features communication platform has led various “internet communities” to spread [10]. Ecotourism and environmental concern communities have sprung up as a result.

Increasingly, the ecological degradation problems are highlighted and environmental protection issues have emerged from social networking sites to express awareness and priority concerns. One of the greatest challenges for sustainable ecotourism is to encourage visitors to act in ways that minimize environmental and experiential impact. This is the case for protected areas where the environment is often fragile and mindful and engaging experiences are sought. Previous research shows that all types of recreational activities can cause environmental damage, even at low use levels [11, 12] and visitor behavior and density levels influence the quality of visitor experiences [13].

Protected area managers employ a suite of strategies to address visitor problems, though for reasons of expediency, cost, and efficiency, education is the preferred and dominant visitor management worldwide [14–16]. As Ham and Weiler [17] pointed out, interpretation is ultimately aimed at communicating messages about a place and, in some instances, the persuasion of people to behave in ways that are consistent with the protected values. Therefore, a better understanding for how visitors think and what factors determine their behavior could contribute to better achieve managerial goals. Given these reasons, we explore the behaviors that are amenable to persuasive influence and how to tap into the relevant thoughts held by visitors about particular actions.

This study is aimed at the most popular social networking sites related to ecotourism issues (ecotourism VCs). We use the technology acceptance model (TAM) proposed by Davis et al. [18] to study IT user intention and behavior to investigate the participation of members of a virtual environmental protection community. Simultaneously, the quality of information and system from the virtual sites to achieve user information and system satisfaction are explored as well as the correlation between user attitude and willingness. In summary, information and system quality, satisfaction, perceived usefulness, and ease of use are discussed. The aforementioned factors affect VC member attitudes, intentions, and participation results. The four research objectives of this study are as follows: (1) integrate two major IT successes related research streams, namely, technology acceptance and user satisfaction; (2) explore the dynamics among object-oriented beliefs and attitudes, behavioral beliefs and attitudes, as well as engagement behavior; (3) investigate the interactive and co-creation value behavior of environmental protection VC; and (4) suggest effective strategies for the promotion of environmental protection issues.

This study determines the motivations and objectives. The remainder of this paper is organized as follows: Section 2 looks at related literature on VC marketing of ecotourism concerns.  Section 3 looks at techniques used and establishes our study framework and decisions for the appropriate research methodology.  Section 4 looks at our online questionnaires for an empirical case study that was distributed and analyzed with statistical software and a series of techniques.  Section 5 shows our findings and the managerial implications are described.  Section 6 is our conclusions and future research directions offered.

## 2. Literature Review

This section describes the relevant literature, including sustainable ecotourism, social network sites and VCs, and related technology acceptance.

The first mention of ecotourism in the English-language academic literature was by Romeril [19]. In the 1990s, the tourism industry realized the profit potential of ecotourism and the suffix “eco” was used by travel agents for marketing. Eventually, ecotourism organizations, such as the International Ecotourism Society (TIES) and the Ecotourism Society (TES), were formed [20]. While some may use the term “ecotourism” as being synonymous with “nature tourism,” “adventure tourism,” “responsible tourism,” “ethical tourism,” and “green tourism;” ecotourism does have specific characteristics that differentiate it from these other segments in the tourism industry. A subset of sustainable tourism (tourism that actively reduces the negative impact of tourism), ecotourism is defined by TIES as “responsible travel to natural areas that conserves the environment and improves the welfare of local people” [21, 22]. Additionally, ecotourism should build a constituency to promote conservation and provide an impetus for private conservation efforts. Under these circumstances, conservation benefits can extend beyond the immediate experience of an ecotourism venture, as ecotourists become active advocates for conservation in the area visited as well as at home [23].

Ecotourism has been identified as a growing niche market for years. According to the World Resources Institute, while tourism grew by 4% in the early 1990s, “nature travel” grew at a rate between 10 and 30% [24]. World Tourism Organization estimates show global spending on the more narrowly defined ecotourism market increasing at a rate of 20% per year, about five times the rate for tourism [24]. However, more striking than this growth is the identification of ecotourism by its advocates as part of an ethical lifestyle (a form of life politics). Particularly, the idea, ecological degradation, is an escalating global threat. Increasingly, the worldwide awareness and concern expressed regarding the environmental problems that surround them. The concern for environmental issues focuses on education as to improve environmental behavior [25] and to study behavior as it relates to remediation of environmental issues. Thus, one way experts can promote environmental improvement through mediators is to examine the link between educational intervention and responsible behavioral change [25]. It is believed that environmental education is linked to environmental behavior. From this, environmental education leads to greater awareness and attitude change that improves environmental protection behavior. Such behavior was successful to change pro-environmental intention and actions that presented as credible environmental information as well as to become actively involved participants in sustainable ecotourism. Sustainable ecotourism has been widely researched in various fields, such as the related literature [26–32].

Some of the latest research on ecotourism, such as analyses on the relationships among tourism [33]; the measurement of sustainable tourism development indicators and developing standards associated [34]; and issues regarding zoos as a morally acceptable form of ecotourism [35]. Our study explores related ecotourism involvement behavior for VC members.

Social networking sites have gained important and vigorous development, most notably Facebook, MySpace, Plurk, and Twitter. The formation of virtual communities from the related literature [36] is an important research area. There were prior studies done regarding social networks. First, House et al. [37] had pointed out that social networks can be divided into two parts: structure and process. As for the structure, they may include social integration and network structure. Yeh and Luo [38] indicated that online dating patterns were similar to the real world. Existent real social networks influenced the formation and development of online communities. Boyd and Ellison [39] claimed that social network sites provided the following functional characteristics: (1) construct a public or semipublic profile within a bounded system; (2) articulated a list of other users with whom they share a connection; and (3) view and traverse their list of connections and those made by others within the system. Xia and Bu [40] described the topic in social network analysis; community detection can help us discover the network properties shared by its members. The VC was widely used for purposes of collaborative recommendation [41], knowledge sharing [42–44], and online market research [45, 46]. Although the nature and nomenclature of these connections may vary from site to site, professional VCs provide spaces that allow domain experts to interact, to assist in the creation, and to share tacit knowledge with the goal of becoming an intelligent enterprise [47–49].

The related technology acceptance literature includes the theory of reasoned action (TRA), theory of planned behavior (TPB), and the technology acceptance model (TAM). TRA was improved by Fishbein and Ajzen [50] and was based on social psychology theory that was widely and successfully applied in various disciplines. TRA states that behavioral intentions to perform a specific behavior predict, explain, or influence actual performance of the said behavior. TPB predicts deliberate behavior based on the assumption that behavior is deliberative and planned [51]. TPB suggests that behavior is determined by intention to perform the behavior and that this intention is, in turn, a function of attitude toward the behavior and subjective norm. TAM proposed by Davis et al. [18] studied the idea of IT user intention and behavior. This model is one of the most acceptable models in the investigation of IT related behaviors. It encompassed Web 2.0 technology focused subjects [52] and recognized two primary principles: “Perceived Usefulness” and “Perceived Ease of Use,” which are major predictors of user attitude and complete emotional reaction for usage.

## 3. Methods and Materials 

### 3.1. Information System Success Model

In the system management area, a crucial topic is to successfully implement an information system (IS) into an organization. An IS was used to enhance a business to create competitive advantage [53]; however, IS of IT will result in failure if it not accepted by its users. Therefore, the evaluation of success of an IS success model (ISSM) has been an important subject to organizations.

DeLone and McLean [54] proposed a successful IS model that indicated the factors that influenced successful IS is comprised of six parts: (1) system quality, (2) information quality, (3) use, (4) user satisfaction, (5) individual impact, and (6) organizational impact. They showed that system and information quality affects the level of use and user satisfaction as well as the level of use to positively or negatively affect user satisfaction. In additional, use level and user satisfaction will affect individuals and organizations. Pitt et al. [55] has added another factor “service quality” in addition to the six listed above. They demonstrate that service quality along with system quality and information quality together affected the use and user satisfaction levels. DeLone and McLean [56] later augmented this revised successful IS model. The revised model additionally added service quality as well as including system quality, information quality, service quality, user intention, user satisfaction, and net benefit.

The literature on user satisfaction indicates that IS characters were core factors, such as system quality, information quality, and service quality in a successful IS model as well as higher order and overall expectancy disconfirmation in the post-acceptance model (PAM) [54, 57]. Bhattacherjee [57] proposed that PAM should be combined with expectancy disconfirmation theory (EDT) and TAM to better investigate user intentions for continual use IS. Furthermore, user satisfaction was regarded as the attitude toward a particular IS, whereas, it should be considered as an object-oriented attitude [58, 59]. This opinion is indicated by various satisfaction correspondence measurements that have adopted system character-oriented measures [60–62].

System characters affect the beliefs and attitudes toward the system itself. Along with behavior beliefs and attitudes as media, they manipulated final use behavior of the system. For example, the perceived reliability of an e-commerce website cannot directly control the use of that website. Nevertheless, it can affect his/her attitude (satisfaction) toward that website and, then, buttress the beliefs (i.e., ease of use) and attitude to use it, and, eventually, the use behavior. Wixom and Todd [59] effectively partitioned and empirically supported the relationship of object-oriented beliefs, object-oriented attitude, behavior beliefs, and behavior attitude based on the expectancy-value theory and the correspondence principle.

### 3.2. The Delphi Method

The Delphi method is a research technique that is used to address complex problems by using a structured communication process of a panel of experts [63] to forecast, make decisions, and solve complex problems. With objective application of the Delphi method, we explore creative ideas and produce valuable information. Knowledge collected during the Delphi study is synthesized and distilled from the use of a series of questionnaires. Responses to questionnaires were collected on site and were reviewed directly [64]. A few of the features of the Delphi method include: (1) rapid consensus, (2) participants can reside anywhere, (3) coverage of wide range of expertise, and (4) avoid groupthink. The limitations of the Delphi method include: (1) cross impact neglected in the original form, (2) does not cope well with paradigm shifts, and (3) success of the method depends on the quality of the participants. Delphi has been applied to various issues, such as to forecast a specific and single-dimension future issue, consensus building, and avoidance of groupthink and to generate creative ideas [63]. The features of the Delphi method provide comprehensive expert opinion and much needed objective consensus.

The latest research on ecotourism uses the Delphi method. For example, they analyze the relationships among tourism [33] and developed a point evaluation system for ecotourism destinations [65]. This study thus uses the Delphi method to analyze VC for eco-travel expert consensus.

In the standard Delphi method, several experts are consulted for estimations of a project or to prognosticate it. The process of Delphi method is described as follows.

1. A project manager prepares a description of the project, in that the individual partial products are listed and prepared on a job form.
2. The project manager presents the goals of the overall project and distributes copies of the job form to each expert; however, it does not take place of a discussion of the estimations.
3. Each expert estimates the work packages contained in the job form; there is no cooperation between experts.
4. All job forms are collected and evaluated by the project manager.
5. If serious discrepancies result, then these are commented on by the project manager uniformly on all job forms as regard to the deviation. Each job form is then returned to its original editor,
6. The experts consider their estimations as a function of the comments.
7. The described loop repeats itself until the estimations independently (in a range of tolerance) consent to adjustment.
8. The average values are calculated and presented by all estimations as the final estimation.

### 3.3. The ISM Method

Warfield [66, 67] first proposed interpretive structural modeling (ISM) to analyze complex socioeconomic systems. It is a process that helps individuals or groups to structure domain knowledge into a model of interrelationships to enhance the understanding of its complexity. The result of the ISM process is represented by a graph that shows the directed relationships as well as hierarchical levels of elements within the system under consideration. A few features of the ISM method include: (1) incorporating the subjective judgments and the knowledge base of experts systematically, (2) to provide ample opportunity for revision of judgments, and (3) computational efforts involved are far less for criteria ranging from 10 to 15 numbers as well as used as a handy tool for real-life applications [68]. The limitations of the ISM method include: (1) the contextual relation among the variables always depends on user knowledge and familiarity with the firm, its operations, and its industry; (2) the bias of the judgment variables influence the final result; (3) ISM acts as a tool to impose order and direction on the complexity of relationships among the variables; and (4) there is no weight associated with the variables [69]. ISM has been widely applied in various fields, such as supply chains [70], balanced scorecard [71], success factors [72], product design [73], and risk analysis [74]. The ISM method can transform nebulous thoughts and ideas into an intuitive model of structural relationships to understand the relationship between the variables.

The computational processes in the ISM method are described in the following steps.

1. Identification of elements through research (e.g., literature review) or expert opinion (e.g., Delphi or brainstorming).
2. Specification of contextual relationship depends on the objective and nature of the case.
3. Construction of a structural self-interaction matrix (SSIM) in four types of possible relationships between the elements (a & b).
4. Transformation of SSIM into an initial reachability matrix (RM) in the rules for the substitution of 1s and 0s.
5. Checking the initial RM for transitivity.
6. Partitioning levels of the final RM.
7. Building ISM digraph and model.

**Figure 1:**
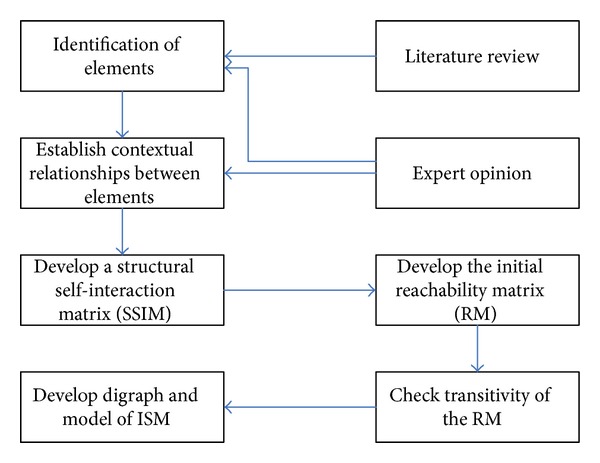
Flow diagram for implementing ISM.

The ISM is generated by replacing all element numbers with the actual element description. Finally, the ISM gives a clear picture of the relationships among the system of elements. Conclusively, Figure 1 shows the above steps of the ISM method. Furthermore, the details of ISM method can be referred to the Warfield [66, 67] for the limited space.

### 3.4. The DEMATEL Method

The decision making trail and evaluation laboratory (DEMATEL) method is a mathematical procedure originated from the Geneva Research Centre of the Battelle Memorial Institute designed to deal with important issues of world societies [75, 76]. The DEMATEL possesses some excellent features. For example, it is based on matrices that represent the contextual relation as well as strength of influence of the elements for the target system. It converts the cause-effect relationship of elements into visible structural models. With its practical benefits, the DEMATEL has been widely applied in various fields, such as marketing [77, 78], education [79, 80], investment [81], supply chain management [82, 83], smart phone [84], and influential factors [85]. The DEMATEL method has advantages that help researchers better understand the nature of the problem.

Mathematically, the procedures of DEMATEL are narrated step-by-step as follows.

**Step 1**

Generate the initial direct-relation matrix. Acquire the assessments about direct affect between each pair of elements from experts. The pair-wise comparison designated by four levels: 0, 1, 2, and 3 to represent “No influence,” “Low influence,” “High influence,” and “Very high influence,” respectively. The initial direct-relation matrix *A* is a *n* × *n* matrix, in which *a*_*ij*_ is denoted as the degree to which the element *i* affects the element *j* is formatted as *A* = [*a*_*ij*_]_*n*×*n*_.

**Step 2**

Normalize the initial direct-relation matrix. The normalized direct-relation matrix *X* = [*x*_*ij*_] can be obtained from (1) and (2).

(1) s=max⁡ [max⁡1≤i≤n∑j=1naij,max⁡1≤j≤n∑i=1naij],         i,j∈{1,2,…n},

(2) $$\begin{array}{c} X=\frac{1}{s}A, \end{array}$$

where (1) represents the maximum values of the sums of all the rows and the sums of all the columns and (2) represents the normalized initial direct-relation matrix. All elements in matrix *X* comply with 0 ≤ *x*_*ij*_ ≤ 1 and all principal diagonal elements are equal to 0.

**Step 3**

Compute the total relation matrix. After Step 2, the total relation matrix, *T*, is obtained by using the following numerical calculation:

(3) T  =X+X2+⋯+Xp=X×(I−X)−1  =  [xij]n×np→∞,

where *p* represents the power. Hence, when *p* tends to infinity, the matrix *X* will converge. Furthermore, *I* is the identity matrix.

**Step 4**

Calculate the sum of rows and columns of matrix *T*. The sum of rows and the sum of columns are separately denoted as vector *D* and vector *R* as follows:

(4) $$\begin{array}{c} T={\left[t_{ij}\right]}_{n\times n},\;i,j=\left\{1,2\ldots ,n\right\},\\ D={\left[\sum_{j=1}^{n}t_{ij}\right]}_{n\times 1}={\left[t_{ij}\right]}_{n\times 1},\\ R={\left[\sum_{i=1}^{n}t_{ij}\right]}_{1\times n}={\left[t_{ij}\right]}_{1\times n}. \end{array}$$

**Step 5**

Construct a cause-effect diagram. The cause-effect diagram is drawn by mapping the data set of the (*D* + *R*, *D* − *R*). The horizontal axis vector (*D* + *R*) named “prominence” is made by adding *D* to *R*, which shows the importance of the element. Similarly, the vertical axis (*D* − *R*) named “relation” is made by subtracting *D* from *R*. When (*D* − *R*) is positive, the element belongs to the cause group; otherwise, the element belongs to the effect group [86, 87].

After calculating the means of (*D* + *R*) and (*D* − *R*), the causal-effect diagram is divided into four quadrants, I to IV. Elements in quadrant I have high prominence and relation which indicates the highest interaction influence level with other elements. Thus, they are identified as driving factors; elements in quadrant II have low prominence but high relation, are identified as voluntariness; elements in quadrant III have low prominence and relation. They are relatively disconnected from the system. The elements in quadrant IV have high prominence and low relation, which indicates their importance as impacted by other elements [88]. From this diagram, the complex interrelationship among elements is visualized to provide valuable insight for decision making. Especially, the DEMATEL method needs experts to decide on a threshold value to concentrate on most important effects from consideration in matrix *T*.

### 3.5. Research Methodology

This section introduces the study procedures, including research framework, research design, and data collection.

#### 3.5.1. Research Framework

**Figure 2:**
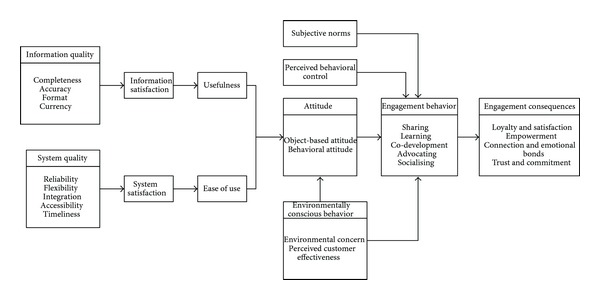
Research framework of this study.

Based on the above related literature review, this study organizes ISSM, TAM, TPB, and TRA models to propose a research framework for exploring member engagement behavior as shown in Figure 2 with 12 major dimensions. In this framework, information quality, system quality, information satisfaction, and system satisfaction are based on ISSM; usefulness, ease of use, and attitude are related to TAM; subjective norms, perceived behavior control, environmentally conscious behavior are constructed from TRA; and engagement behavior, as well as engagement consequences are involved in TRA. These dimension will be measured by expert opinion through Delphi technique as described later.

#### 3.5.2. Research Design

**Figure 3:**
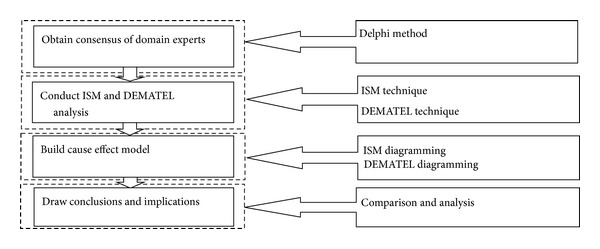
Flowchart of the proposed method of the study.

Since members of environmental protection VCs exert a multi-function dynamic, complex, and value co-creation behavior, multi-criteria decision making (MCDM) is employed to gain insights when concerned with the relationships. This study presents ISM and DEMATEL methods to solve real-life applications of MCDM problems, which provide a complete understanding of the procedures of MCDM tools.  Figure 3 shows a flowchart of our proposed method of study.

#### 3.5.3. Data Collection

This subsection briefly elucidates the computational processes using an empirical case study, including case introduction, research subject definition, and instrument development.

(*1)  Case Introduction. *Two most popular ecotourism VCs in Taiwan, EZTravel and LulalaTravel, are committed to ecological and environmental protection.

First, for a constructed web site, http://eztravel.com/ was established in January 2000 to provide a full range service of online booking and online payments. It has total capital of NT$218 million with 460 employees and more than 2.2 million served members (many might be tourists). It has been the leader among domestic online travel agents in Taiwan. It maintained sustainable rapid growth in the revenue and has been ranked a top operating performer among domestic tourism websites. http://eztravel.com/ has aimed to aggressively develop differentiated fashionable products to satisfy various demands from consumers. It enhanced its core competitiveness to create profitability. EZTravel has originated the following specialized tours: environmental protection tourism, luxury tours on trains, million dollars around the world, tours of most of the world, international travelers, and a variety of tours with local themes. Particularly, environmental protection tourism has been welcomed and focused on.

Second, LulalaTravel Company was established on December 2005. The company has a mission to serve younger travelers, which has originated from the China Youth Corp. It has been expanding domestic tourist activities legally and professionally. In April 2006, the company established eight branch offices: Keelung City, New Taipei City, Taichung City, Changhua County, Yunlin County, Chiayi County, Tainan City, and Yilang County. LulalaTravel has 32 professional operators who handle all the business related to tourism as well as offers courses to educate professional tour guides, military instructors, and university staff. Thus, they created sequential training courses for the continual education of tourism professionals. LulalaTravel emphasizes ecotourism based on a mission from the China Youth Corp to provide a variety of services. They have originated many activities, such as educational group athletic activities, mountain training, casual weekends, holiday tours, potential development, upstream canoeing, and survival games. During summer and winter breaks, LulalaTravel offered various educational tasks to perform ecological leisure activities that had incorporated ecological themes for Taiwan.

(*2) Research Subjects. *Twelve professionally active members of ecotourist VCs were invited to engage in this study, mainly by contributing their opinions regarding the relationships among all research variables (Figure 2) through the Delphi process to find group consensus as input to all later related analyses.

(*3) Instrument Development.* Table 1 shows the instruments of this study as constituted by the components and their related elements.

## 4. Experiment and Data Analysis 

**Figure 4:**
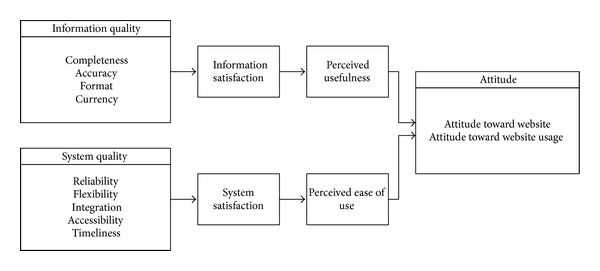
The structure of proposed model 1.

**Figure 5:**
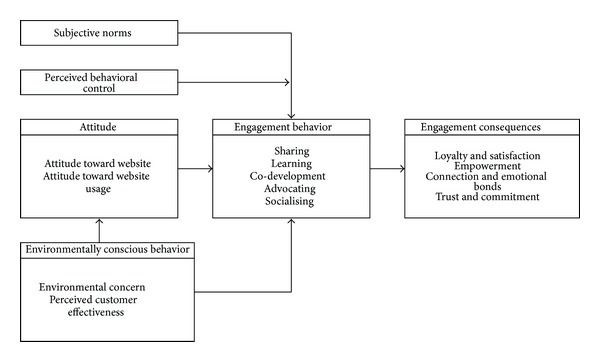
The structure of proposed model 2.

**Figure 6:**
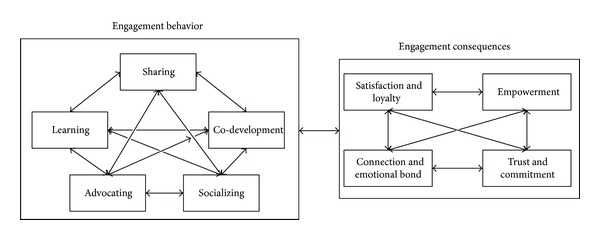
The structure of proposed model 3.

We decomposed our study framework into three models, namely models 1–3 (Figures 4, 5, and 6) to thoroughly investigate the dynamics of the variables identified in our research model. Afterwards, we describe the profile of the experts that we have interviewed; and, then, conduct ISM and DEMATEL analyses for the three models.

### 4.1. Expert Profiles

We conducted a questionnaire to gather the opinions of experts. A total of 12 eco-travel experts participated in our questionnaire using the Delphi method. Thus, 12 surveys were received. The survey lasted from April 2012 until May 2012. The first round of surveys was received on April 16 2012. We compiled and summarized different opinions from the experts and then sent them a second round of questionnaires. After five rounds of opinion consolidation, we received a final consensus from the experts on May 25 2012.  Table 2 summarizes the demographic profile of 12 experts.

### 4.2. ISM Analysis

In ISM analysis, a four-stage approach was used to systematically analyze. They are as follows: (1) construct structural self-interaction matrix, (2) generate reachability matrix, (3) partition the levels, and (4) building the ISM model.

#### 4.2.1. Construct Structural Self-Interaction Matrix

The first step of ISM analysis was to perform analysis on the contextual relationship of variables. Based on the consensus from the expert panel, we captured the relationships in structural self-interaction matrixes (SSIM) for models 1–3.

#### 4.2.2. Generate Reachability Matrix

Next, the SSIM is transformed into a binary matrix called initial reachability matrix by substituting the arrows by related 1 and 0.

#### 4.2.3. Partition the Levels

From the final reachability matrix, the reachability set, and antecedent set for each variable were obtained. The reachability set includes variables themselves and others that help, while the antecedent set consists of variables and the other variables that help. Consequently, the intersection of these sets was derived for all variables. The variable for which its reachability was set to equal its intersection set is identified as the top-level variable in the ISM hierarchy. One important feature of the top-level variable in the hierarchy is that it does not help achieve any other variable above its own level. Therefore, once the top-level variable is identified, it is separated from the other variables. The same process is repeated to find out the next level until the level of each variable was found. Tables 3, 4 and 5 for models 1–3, respectively, show and summarize the results for the iteration process. Particularly, models 1, 2, and 3 show levels of 4, 3, and 2, respectively.

#### 4.2.4. Building the ISM Model

**Figure 7:**
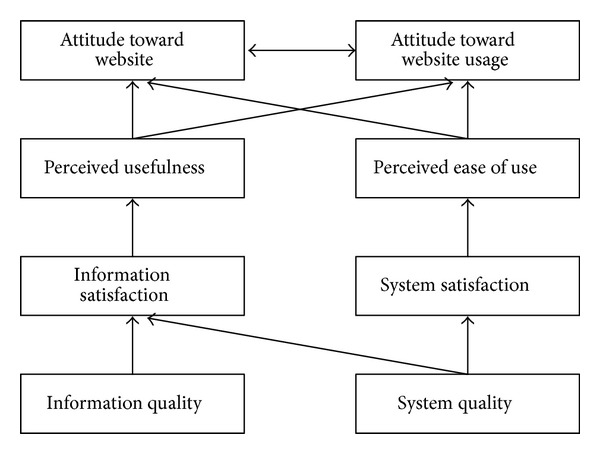
Four levels of structural model of model 1.

**Figure 8:**
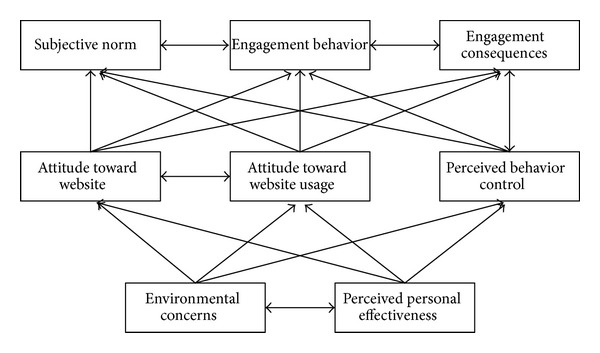
Three levels of structural model of model 2.

**Figure 9:**
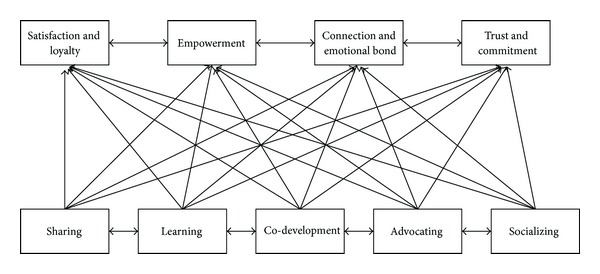
Two levels of structural model of model 3.

Based on the final reachability matrix, the structural model of the proposed three models can be generated. If there is a relationship between variable *i* and *j*, then an arrow is drawn to connect the two points. This graph is called a directed graph or digraph. After removing the transitivity, the digraph is finally transformed into the ISM-based model (Figures 7, 8, and 9) for model(s) 1–3, respectively.

### 4.3. DEMATEL Analysis

As for DEMATEL analysis, the five-stage approach was analyzed in detail and includes: (1) obtain average matrix from experts; (2) normalize the average matrix to get initial direct-relation matrix; (3) compute the total relation matrix; (4) calculate the sum of rows and columns of total relation matrix; and (5) construct the cause-effect diagram.

#### 4.3.1. Obtain Average Matrix from Experts

First, the assessments of the direct affect between each pair of variables designated by the four levels, 0–4, are summarized as an average matrix of models 1–3 (Tables 6, 7, and 8).

#### 4.3.2. Normalize the Average Matrix to Get Initial Direct-Relation Matrix

The average matrix is normalized by dividing all elements by the maximum values from the sums of all rows as well as the sums of all columns. For the limited space, only Table 9 shows the above results on the initial direct-relation matrix of model 1.

#### 4.3.3. Compute the Total Relation Matrix

The direct relation matrix has further raised its power to gain a convergent total relation matrix. For the limited space, only Table 10 shows the above results on the total relation matrix of model 1.

#### 4.3.4. Calculate the Sum of Rows and Columns of Total Relation Matrix

Let vectors *D* and *R* denote the sum of rows and the sum of columns from total relation matrix, respectively, and then the values were obtained for models 1–3. Particularly, the average values of (*D* + *R*) and (*D* − *R*) were taken as the axial cross of *Y* (*D* − *R*) and *X* (*D* + *R*) in the next stage.

#### 4.3.5. Construct the Cause-Effect Diagram

**Figure 10:**
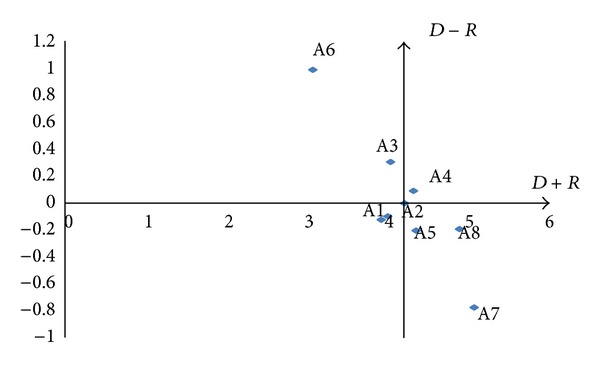
Cause-effect diagram of model 1.

**Figure 11:**
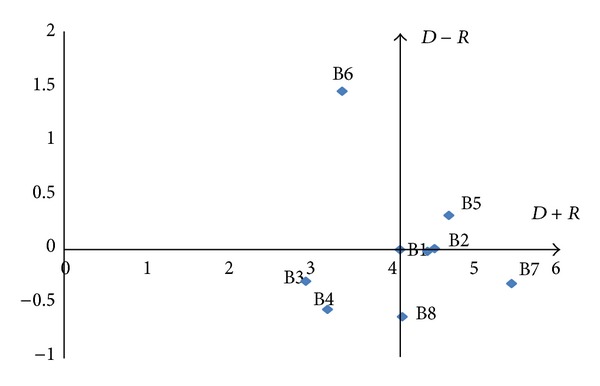
Cause-effect diagram of model 2.

**Figure 12:**
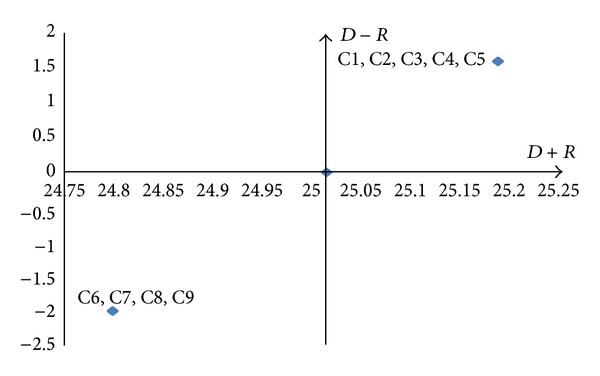
Cause-effect diagram of model 3.

By mapping the data set of (*D* + *R*,  *D* − *R*), a casual diagram was drawn, where *X* of (*D* + *R*, prominence) was made by adding *D* to *R*, and *Y* of (*D* − *R*,relation) was made by subtracting *D* from *R*. Based on the means of (*D* + *R*) and (*D* − *R*), four quadrants were identified with their respective natures. Quadrants I and IV are defined as strong “cause” and “effect” factors of desired outcome, respectively. In contrast, Quadrants II and III are defined as weak “cause” and “effect” factors for desired outcomes, respectively. Thus, the cause-effect diagrams for the Models 1–3 can be drawn as Figures 10, 11, and 12.

## 5. Results and Discussion

We explored the analytical results to mine hidden information from our empirical case study for the research models. The following implied study findings and implications that suggest the management of engagement behavior that stem from the VC are compiled. They are of value to the academics and practitioners who focus on ecotourism community fields.

### 5.1. Findings

**Figure 13:**
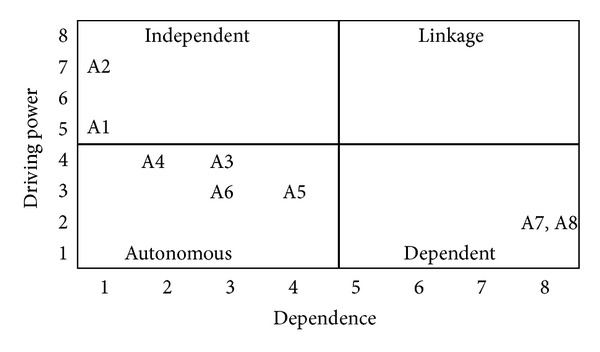
Driving power-dependence diagram of model 1.

**Figure 14:**
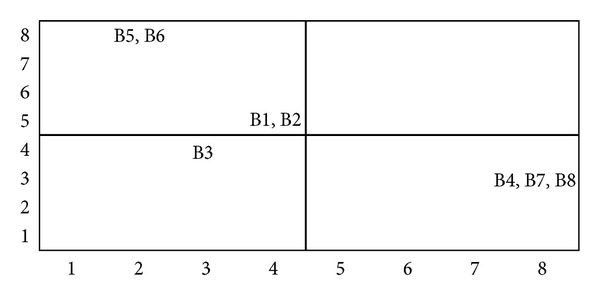
Driving power-dependence diagram of model 2.

**Figure 15:**
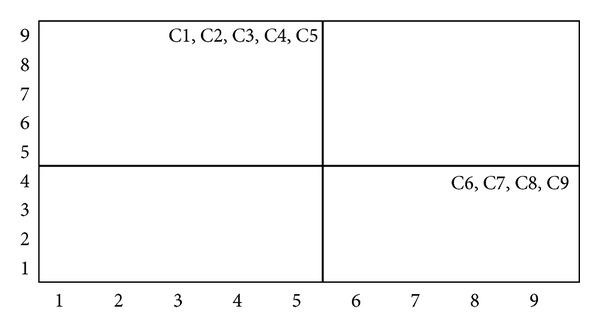
Driving power-dependence diagram of model 3.

(*1) Ecotourism Virtual Community Acceptance*. Given its huge investments and great impact on businesses, the evaluation of information system success has gained wide attention and involvement from academia. Traditionally, this area has been investigated within two primary research streams: the user satisfaction literature of ISSM (e.g., DeLone & McLean [54]) and the technology acceptance literature of TAM (e.g., Davis et al. [18]). These two approaches have been developed in parallel and have not been reconciled or integrated [59]. To bridge this gap, Wixom and Todd [59] have proposed an integrated research model that distinguishes between object-based beliefs and attitudes toward “the system” as well as behavioral beliefs and attitudes toward “using the system” and successfully links two dominant approaches. They also emphasized the correspondence principle for accurate predictions, beliefs, and attitudes must be specified in a manner that is consistent in time, target, and context with the behavior of interest [50]. Namely, to predict IS acceptance behavior, behavioral beliefs and attitudes performed better than object-based beliefs and attitudes. In our model 1 ISM model, all these propositions were supported well. Wixom and Todd [59] validated our study findings. 

(*2) Ecotourism Virtual Community Engagement consequence*. One important track of this study is customer engagement (CE). According to Brodie et al. [89], in the highly dynamic and interactive business environment, CE plays a role in co-creating customer experiences that values and receives increasing attention from business practitioners and academics alike. As salient evidence for this development, Marketing Science Institute (MSI) chose CE as a key research priority for 2010–2012. Even though most CE research is business-oriented, this study assumed the concept also applied to ecotourism VC context. The results of model 3 justified this point.  Table 11 offers justification of the 5 fundamental propositions (FP) of CE summarized by Brodie et al. [89] as well as evidence from this study. 

(*3) Ecotourism Virtual Community Engagement Behavior*. Kim and Han [91] have tested and modified the TPB by adding two important concepts: environmental concerns and perceived customer effectiveness, which contribute to environmentally conscious behavior that helps to critically predict eco-friendly consumer behaviors. This study follows this approach in Model 2 and verifies these two constructs exert critical driving power to impact users' attitude toward the website as well as its usage. Besides, they affect perceived behavioral control directly, then engagement behavior and engagement consequences. Compared with TPB, model 2 revealed an interesting finding that subjective norms play the role of an effect instead of a cause variable. This phenomenon indicates attitudes toward ecotourism VCs and perceived behavioral control work together to shape a social pressure that will encourage engagement. 

(*4) Summarization of Cause and Effect Factors*. Salimifard et al. [93] has suggested a driving power-dependence diagram to help classify various decision factors into four clusters. The cluster in quadrant I include “linkage” elements that have strong driving power and dependence. The implication is that all the factors above this level are affected by them, while these elements are also dependent on lower level factors for the ISM model. The cluster in quadrant II consists of dependent factors that have weak driving power but strong dependencies. Factors in this cluster are the most important and influential ones. The cluster in quadrant III includes “autonomous” factors that have weak driving power and weak dependence. These factors are relatively disconnected from the system. Finally, the cluster in quadrant IV includes “dependent” factors that have weak driving power but strong dependence. These factors are representative of a desired system of outcomes. Figures 13, 14, and 15 show the driving power and dependence diagrams of this study.

(*5) Overlapping Extension of Cause and Effect Factors*. Afterwards, we compared cause and effect variables in models 1–3 identified by different ISM and DEMATEL algorithms. Tables 12 and 13 show the results. Obviously, great overlapping between them, which are highlighted in black-frame, was found. This phenomenon revealed that the performance of two intelligent methods provides similar analytical results toward an exploration of engagement behavior of ecotourism VC members to imply that the study results can be trusted.

Finally, the two methods are desirable for the following two reasons.

*Reasons on Selected Used Techniques.* First, ISM and DEMATEL under the MCDM condition are two major methodologies with the capability to clarify complex relationships between the elements involved in complex decision making. There are several similarities between them, such as they emphasize a cause-effect relationship among several decision elements (e.g., the driving power and dependence in ISM and the prominence and relation in DEMATEL) as well as present the relationships in easily understood diagrams. Nevertheless, ISM considered four possible relationships, while DEMATEL investigated the relationships deeper with a more sophisticated evaluation (from 0 to 4), as well as allowing different degrees of mutual influences. Therefore, ISM is more macro-oriented and DEMATEL more micro-oriented. They can complement each other to exert synergic benefits. As far as our limited knowledge, no research has adopted this approach. We also proved the adequacy of this approach.

### 5.2. Managerial Implications

The management implications in this study are based on the ISM and DEMATEL methods.

#### 5.2.1. ISM Method

(*1)  Model 1*. System and information quality are important factors in IS. Good quality systems bring community members a better site experience and good information quality makes them willing participants in ecotourism VCs for getting the latest ecotourism information.

(*2) Model 2*. Environmental concerns affect the attitude of the community members and ease of design of the system interface in the use of IS. Furthermore, the power of the community is a driving force to learn and share engagement behavior to promote environmental awareness. Ecotourism experiences share common topics of the natural environment through social networking sites.

(*3) Model 3*. The engagement on the public discussion that has enough emotion to form a network of human relationships with certain characteristics of social organization for the promotion of ecotourism VC issues is a positive relationship over time. Thus, common ecotourism virtual issues were developed through learning and sharing among the community members. Particularly, member loyalty enhanced the community and resulted in the real value of the community sites.

#### 5.2.2. DEMATEL Method

(*1) Model 1*. System quality is a major driving power for other system components. Besides, system satisfaction and information satisfaction greatly influence system beliefs and attitude. Consequently, enhancing system infrastructure, such as hardware and software, as well as encouraging information sharing will be beneficial for community members' active and better engagement.

(*2) Model 2*. Environmental concerns and perceived personal effectiveness have strong influencing power on other system components. The emphasis on environmental protection was enhanced through the understanding of the importance of environmental issues. More importantly, the use of VCs to strengthen the basic concepts of international conservation and sustainable development are interesting issues that combine the power of the community members on the internet and the awareness of environmental protection to every corner of the world.

(*3) Model 3*. Sharing, learning, co-development, advocating, and socializing have a strong relationship with other system components. Community members that share their ideas provide mutual understanding as well as contribute to the development of the community to achieve a vested emotion. The relationship and the sharing of member demands are to foster a sense of trust and commitment. The operation of the community website provides the members a centripetal force to achieve the maximum benefits of the community network.

## 6. Conclusions 

The MCDM is a sub-disciplinet of operations research that explicitly considers multiple criteria in decision-making environments. However, there typically exist multiple conflicting criteria that need to be evaluated for making decisions. Therefore, structuring complex problems well and considering multiple criteria explicitly lead to more informed and better decisions. They are developed methods that transform such complex problem into essentially single criterion problems. They are used to solve MCDM problems by constructing value functions. Perhaps, a well-known method includes ISM and DEMATEL. Particularly, the two methods have seldom been seen in hybrid use to solve MCDM problems in ecotourism VCs.

Given the above reasons, this study focused on filling these knowledge gaps and conducting an intelligent hybrid model to solve a real life application problem that involves MCDM toward sustainable ecotourism. This study has proposed hybrid expert-based ISM and DEMATEL models that are suitable for those members of environmental protection VCs who intend to use intelligent systems. This study performs well and provides useful insight into the key characteristics that explores member engagement behavior in the VC industry and is critical with respect to responding to the rapidly changing environment under VC members that exert the multi-functions of dynamic, complex, and value co-creation behavior. The analytical results have important implications that are worthwhile for practitioners and academics that focus on environmental protection in VCs. Moreover, future research can be done in three directions as follows. (1) To screen and organize a domain expert panels with excellent experience in ecotourism virtual communities and apply procedures suggested by Delphi method and focus groups to find consensus efficiently and effectively from the use of larger samples to conduct structural equation modeling (SEM) to cross-verify the study results; (2) to investigate the dynamic nature decision elements more insightfully; analytical network process (ANP) can be applied especially for the engagement related variables; and (3) other qualitative research methods, such as interactive qualitative analysis (IQA) and means-ends chain (MEC) by laddering analysis can also be applied to gain more detailed responses from domain experts.

## Figures and Tables

**Table 1:** System component definitions.

Components	Definitions	Elements	Reference
C1: engagement consequences	The desired results of engagement behavior	E11: loyalty and satisfaction E12: empowerment E13: connection and emotional bonds E14: trust and commitment	[89]

C2: engagement behavior	The interaction and value co-creation behavior of community members	E21: sharing E22: learning E23: co-development E24: advocating E25: socializing	[89]

C3: perceived behavioral control	An individual's perception on the ease or difficulty of conducting the behavior	—	[51, 90]

C4: attitude	A favorable or unfavorable evaluation of something	E41: object-based attitude E42: behavioral attitude	[59]

C5: environmentally Conscious Behavior	Recognizing the serious of environmental problems, people proactively engaged in recycling, saving electricity and water, and so forth	E51: environmental concern E52: perceived customer effectiveness	[91]

C6: subjective norms	An individual's estimate of the social pressure on him/her to engage or not engage in the target behavior	—	[51]

C7: perceived usefulness	Beliefs concerning instrumental outcomes associated with technology use	—	[92]

C8: perceived ease of use	Beliefs that technology use will be relatively free of cognitive burden	—	[92]

C9: information satisfaction	The degree of favorableness with respect to the information produced by the system	—	[59]

Ca: system satisfaction	The degree of favorableness with respect to the system and the mechanics of interaction	—	[59]

Cb: information quality	Desired features of the information produced by the system	Cb1: completeness Cb2: accuracy Cb3: format Cb4: currency	[59]

Cc: system quality	Desired features of the system and the mechanism of interaction	Cc1: reliability Cc2: flexibility Cc3: integration Cc4: accessibility Cc5: timeliness	[59]

**Table 2:** The demographic variables of 12 experts.

Demographic variables	Number (*N* = 12)	Percentage
Gender		
Male	5	41.7%
Female	7	58.3%
Age		
Below 30 years old	3	25.0%
31~40	3	25.0%
41~50	4	33.3%
51 years old and above	2	16.7%
Educational		
Certificate	2	16.7%
Bachelor	7	58.3%
Master	2	16.7%
Doctoral	1	8.3%
Experience in online tourism		
Less than 5 years	2	16.7%
5–10	3	25.0%
11–15	1	8.3%
16–20	4	33.3%
21 years and above	2	16.7%
Ecotourism virtual communities (most frequently engaged)		
EZTravel	6	50.0%
LulalaTravel	6	50.0%

**Table 3:** Levels of model 1 elements.

Element	Reachability set	Antecedent set	Intersection set	Level
A1	A1A3A5A7A8	A1	A1	4
A2	A2A3A4A5A6A7A8	A2	A2	4
A3	A3A5A7A8	A1A2A3	A3	3
A4	A4A6A7A8	A2A4	A4	3
A5	A5A7A8	A1A2A3A5	A5	2
A6	A6A7A8	A2A4A6	A6	2
A7	A7A8	A1A2A3A4A5A6A7A8	A7A8	1
A8	A7A8	A1A2A3A4A5A6A7A8	A7A8	1

**Table 4:** Levels of model 2 elements.

Element	Reachability set	Antecedent set	Intersection set	Level
B1	B1B2B4B7B8	B1B2B5B6	B1B2	2
B2	B1B2B4B7B8	B1B2B5B6	B1B2	2
B3	B3B4B7B8	B3B5B6	B3	2
B4	B4B7B8	B1B2B3B4B5B6B7B8	B4B7B8	1
B5	B1B2B3B4B5B6B7B8	B5B6	B5B6	3
B6	B1B2B3B4B5B6B7B8	B5B6	B5B6	3
B7	B4B7B8	B1B2B3B4B5B6B7B8	B4B7B8	1
B8	B4B7B8	B1B2B3B4B5B6B7B8	B4B7B8	1

**Table 5:** Levels of model 3 elements.

Element	Reachability set	Antecedent set	Intersection set	Level
C1	C1C2C3C4C5C6C7C8C9	C1C2C3C4C5	C1C2C3C4C5	2
C2	C1C2C3C4C5C6C7C8C9	C1C2C3C4C5	C1C2C3C4C5	2
C3	C1C2C3C4C5C6C7C8C9	C1C2C3C4C5	C1C2C3C4C5	2
C4	C1C2C3C4C5C6C7C8C9	C1C2C3C4C5	C1C2C3C4C5	2
C5	C1C2C3C4C5C6C7C8C9	C1C2C3C4C5	C1C2C3C4C5	2
C6	C6C7C8C9	C1C2C3C4C5C6C7C8C9	C6C7C8C9	1
C7	C6C7C8C9	C1C2C3C4C5C6C7C8C9	C6C7C8C9	1
C8	C6C7C8C9	C1C2C3C4C5C6C7C8C9	C6C7C8C9	1
C9	C6C7C8C9	C1C2C3C4C5C6C7C8C9	C6C7C8C9	1

**Table 6:** The average matrix of model 1.

	A1	A2	A3	A4	A5	A6	A7	A8	Row total
A1	0	1	4	1	3	1	2	2	14
A2	1	0	1	4	1	3	2	2	14
A3	3	1	0	1	4	1	3	3	16
A4	1	3	1	0	1	4	3	3	16
A5	2	1	3	1	0	1	4	4	16
A6	1	2	1	3	1	0	4	4	16
A7	1	1	2	2	3	3	0	4	16
A8	1	1	2	2	3	3	4	0	16

Column total	10	10	14	14	16	16	22	22	22

**Table 7:** The average matrix of model 2.

	B1	B2	B3	B4	B5	B6	B7	B8	Row total
B1	0	4	1	1	3	3	4	3	19
B2	4	0	1	1	3	3	4	3	19
B3	1	1	0	1	1	1	4	3	12
B4	1	1	1	0	1	1	4	3	12
B5	4	4	1	1	0	4	4	3	21
B6	4	4	1	1	4	0	4	3	21
B7	3	3	3	3	3	3	0	4	22
B8	2	2	2	2	2	2	2	0	14

Column total	19	19	10	10	17	17	26	22	26

**Table 8:** The average matrix of model 3.

	C1	C2	C3	C4	C5	C6	C7	C8	C9	Row total
C1	0	4	4	4	4	4	4	4	4	32
C2	4	0	4	4	4	4	4	4	4	32
C3	4	4	0	4	4	4	4	4	4	32
C4	4	4	4	0	4	4	4	4	4	32
C5	4	4	4	4	0	4	4	4	4	32
C6	3	3	3	3	3	0	4	4	4	27
C7	3	3	3	3	3	4	0	4	4	27
C8	3	3	3	3	3	4	4	0	4	27
C9	3	3	3	3	3	4	4	4	0	27

Column total	28	28	28	28	28	32	32	32	32	32

**Table 9:** Initial direct-relation matrix of model 1.

	A1	A2	A3	A4	A5	A6	A7	A8
A1	0.000	0.045	0.182	0.045	0.136	0.045	0.091	0.091
A2	0.045	0.000	0.045	0.182	0.045	0.136	0.091	0.091
A3	0.136	0.045	0.000	0.045	0.182	0.045	0.136	0.136
A4	0.045	0.136	0.045	0.000	0.045	0.182	0.136	0.136
A5	0.091	0.045	0.136	0.045	0.000	0.045	0.182	0.182
A6	0.045	0.091	0.045	0.136	0.045	0.000	0.182	0.182
A7	0.045	0.045	0.091	0.091	0.136	0.136	0.000	0.182
A8	0.045	0.045	0.091	0.091	0.136	0.136	0.182	0.000

**Table 10:** Total relation matrix of model 1.

	A1	A2	A3	A4	A5	A6	A7	A8
A1	0.189	0.222	0.303	0.209	0.310	0.081	0.312	0.267
A2	0.223	0.197	0.192	0.344	0.229	0.169	0.319	0.273
A3	0.330	0.253	0.161	0.247	0.372	0.089	0.382	0.329
A4	0.252	0.338	0.218	0.206	0.245	0.220	0.386	0.333
A5	0.279	0.227	0.292	0.214	0.196	0.084	0.403	0.371
A6	0.221	0.277	0.189	0.298	0.235	0.040	0.398	0.366
A7	0.241	0.247	0.235	0.284	0.330	0.175	0.257	0.373
A8	0.279	0.283	0.269	0.303	0.352	0.178	0.457	0.221

**Table 11:** The justification of research results on FP of CE.

	Fundamental propositions	Justification
FP1	CE reflects a psychological state that occurs by virtue of interactive customer experiences with a focal agent/object within specific service relationships.	(i) The focal agent/object a customer interacts with may be a brand, product, or organization (i.e., the ecotourism VC). (ii) Focal CE behaviors that have a brand- or firm-focus extend beyond transactions/purchase (the environmental concern in ecotourism VC transcends over transactions). (iii) Two-way interactions generating CE may occur within a broader network of customers, stakeholders, and other actors in specific service relationships (there are different participating roles in ecotourism VC).

FP2	CE states occur within a dynamic, iterative process of a service relationship that co-creates value.	CE processes may range from short-term to long-term, relatively stable to highly variable processes typified by CE levels varying in complexity over time (there is different degrees of involvement in ecotourism VC).

FP3	CE plays a central role within a nomological network of service relationships.	(i) There is antecedent as well as consequence factors of ecotourism virtual community engagement behavior, as shown in our research framework. (ii) The mutual-influence and iterative nature of engagement behaviors imply that specific CE consequence may extend to be an antecedent in the next process.

FP4	CE is a multidimensional concept subject to a context and/or stakeholder-specific expression of relevant cognitive, emotional, and behavioral dimensions.	(i) Variables in this research framework include cognitive (e.g., information quality and system quality), emotional (e.g., attitude toward website itself and website usage), and behavioral (e.g., engagement behavior) dimensions in nature. (ii) Different situational conditions might generate distinct CE complexity levels.

FP5	CE occurs within a specific set of situational conditions generating differing CE levels.	Specific interaction between a customer and a focal agent/object and other actors within specific focal relationships may generate different levels of cognitive, emotional, and/or behavioral CE intensity, depending on specific CE stakeholder and contextual contingencies driving particular CE levels (the casual model acquired by ISM or DEMATEL analyses show this dynamic well).

**Table 12:** Cause and effect factors identified by ISM model.

Model	Dependent	Independent
1	A7 (attitude toward website)	A1 (information quality)
	A8 (Attitude toward website usage)	A2 (system quality)

2	B4 (subjective norm)	B1 (attitude toward website)
	B7 (engagement behavior)	B2 (attitude toward website usage)
	B8 (engagement consequences)	B5 (environmental concerns)
		B6 (perceived personal effectiveness)

3	C6 (satisfaction and loyalty)	C1 (sharing)
	C7 (empowerment)	C2 (learning)
	C8 (connection and emotional bond)	C3 (co-development)
	C9 (trust and commitment)	C4 (advocating)
		C5 (socializing)

**Table 13:** Cause and effect factors identified by DEMATEL model.

Model	Strong dependent variables	Strong independent variables
1	A5 (perceived usefulness)	A4 (system satisfaction)
	A7 (attitude toward website)	
	A8 (attitude toward website usage)	

2	B1 (attitude toward website)	B2 (attitude toward website usage)
	B7 (engagement behavior)	B5 (environmental concerns)
	B8 (engagement consequences)	

3	C6 (satisfaction and loyalty)	C1 (sharing)
	C7 (empowerment)	C2 (learning)
	C8 (connection and emotional bond)	C3 (co-development)
	C9 (trust and commitment)	C4 (advocating)
		C5 (socializing)
